# Cylindrically bent Laue analyzer in an X-ray Raman/emission spectrometer: performance tests and a comparison with spherically bent Bragg analyzers

**DOI:** 10.1107/S1600577524010634

**Published:** 2025-01-01

**Authors:** Nozomu Hiraoka

**Affiliations:** a National Synchrotron Radiation Research Center Hsinchu30076 Taiwan; Bhabha Atomic Research Centre, India

**Keywords:** X-ray Raman scattering, X-ray emission spectroscopy, Laue analyzers, spherically bent Bragg analyzers, cylindrically bent Laue analyzers

## Abstract

The performances of a spherically bent Bragg analyzer and a cylindrically bent Laue analyzer in an X-ray Raman/emission spectrometer are compared. The reflectivity and energy resolution are evaluated from the intensity of the elastic scattering and the width of the energy distribution on a SiO_2_ glass sample. Widely used, Bragg analyzers display excellent performance at the photon energy *E* ≤ 10 keV. However, at higher *E*, the reflectivity and the resolution gradually deteriorate as *E* increases, showing poor performance above 20 keV. On the other hand, the reflectivity of the Laue analyzer gradually increases at *E* > 10 keV, displaying excellent reflectivity and good resolution around 20 keV. The Laue analyzer is suitable for X-ray absorption spectroscopy in high-energy-resolution fluorescence-detection mode or X-ray emission spectroscopy on 4*d* transition metal compounds. Furthermore, the X-ray Raman features of the lithium *K*-edge in LiF and the oxygen *K*-edge feature in H_2_O, measured by nine Bragg analyzers (2 m radius) at *E* ≃ 9.9 keV and by five Laue analyzers (1.4 m radius) at *E* ≃ 19.5 keV, have been compared. Similar count rates and resolutions are observed.

## Abstract 2

The performances of a spherically bent Bragg analyzer and a cylindrically bent Laue analyzer in an X-ray Raman/emission spectrometer are compared.

## 1. Introduction

Spherically bent crystal analyzers are widely utilized in X-ray Raman/emission spectrometers (Schülke, 2007 ▸; Verbeni *et al.*, 2009 ▸; Sahle *et al.*, 2015 ▸, 2023 ▸; Huotari *et al.*, 2017 ▸; Moretti Sala *et al.*, 2018 ▸; Glatzel *et al.*, 2021 ▸; Edwards *et al.*, 2022 ▸). When the Rowland condition is fulfilled – namely, the sample (or the light source on it) and the detector are placed on a circle tangential to the crystal analyzer with a diameter equal to the bending radius of the crystal – photons emitted from the source are monochromated by the analyzer and then focused into a spot on the detector. Generally, for a spherically bent analyzer, a large Bragg angle reflection is exploited to achieve high energy resolution, high reflectivity and a large solid angle. However, as shown below, it becomes more difficult to maintain such advantages as the photon energy *E* increases. To maintain the large Bragg angle, it is necessary to utilize a very high index reflection, which generally has a small scattering form factor and thus a narrow Darwin width. A narrow Darwin width is essential to achieve an ultra-high resolution (1–10 meV). Nonetheless, the resolution generally required for X-ray Raman scattering (XRS) or X-ray emission spectroscopy (XES) is in the range 0.1–1 eV, and thus a too-small scattering form factor is disadvantageous in terms of the reflectivity or the integrated reflectivity. On the other hand, in the Laue geometry, X-rays transmit through the analyzer interior, resulting in low reflectivity or intensity of the reflected beam at *E* ≤ 10 keV due to absorption. Nevertheless, it exhibits performance suitable for XRS and XES at higher energies as discussed in our previous reports (Hiraoka *et al.*, 2013 ▸, 2016 ▸). The author and co-workers have continuously studied the application of Laue analyzers since then (Nyrow *et al.*, 2014 ▸; Hagiya *et al.*, 2020 ▸; Hiraoka *et al.*, 2023 ▸), and, currently, a spectrometer equipped with five Laue analyzers and five detectors are under commissioning. Although there is no question that the Bragg analyzer shows greater performance at *E* ≤ 10 keV (Cai *et al.*, 2005 ▸; Gordon *et al.*, 2007 ▸; Mao *et al.*, 2010 ▸; Huotari *et al.*, 2011 ▸; Willers *et al.*, 2012 ▸; Sahle *et al.*, 2013 ▸; Hiraoka *et al.*, 2015 ▸; Georgiou *et al.*, 2019 ▸, 2022 ▸), it may be still a question of whether the Laue analyzer indeed exhibits great performance at higher energies (Ravel *et al.*, 2018 ▸; Jagodziński *et al.*, 2019 ▸). In this article, we report on the (i) performance tests of the Bragg and Laue analyzers with the elastic scattering measurements on an SiO_2_ glass sample, (ii) X-ray absorption spectroscopy (XAS) measurements in high-energy-resolution fluorescence-detection (HERFD) mode on 4*d* transition metals by the bent Laue analyzer, and (iii) a comparison between X-ray Raman features of the Li *K*-edge in LiF and O *K*-edge features in water measured by the two different analyzers.

## 2. Performance test with the elastic scattering in SiO_2_ glass

To evaluate the performance of the two types of analyzers, the energy profile of elastic scattering was measured on an SiO_2_ glass sample. The intensity and the width give the reflectivity and the instrumental resolution, respectively. The experiments were performed at the Taiwan inelastic X-ray scattering beamline (BL12XU) at SPring-8 (Cai *et al.*, 2004 ▸). Synchrotron radiation emitted from an undulator light source was monochromated at *E* = 9.9–25.9 keV with an Si 111 double-crystal monochromator (DCM). The energy width of an Si 111 DCM is generally d*E*/*E* = 1.4 × 10^−4^ over *E* = 9.9–25.9 keV (LR setup). Another setup, where a high-resolution post monochromator was installed after the DCM, was also tried for a more critical test (HR setup). It is expected from analysis on a DuMond diagram that Si 220 channel-cut crystals in four-bounce mode give an energy width of d*E*/*E* = 0.3 × 10^−4^ over the current *E* range (Matsushita & Hashizume, 1983 ▸). The beam was focused to a 15 µm × 30 µm spot on the sample (V × H) by a Pt-coated Kirkpatrick–Baez mirror (Huang *et al.*, 2008 ▸).

The energy profile of the elastic scattering on the SiO_2_ glass sample (20 mm × 20 mm surface and 0.3 mm thickness) was measured by a spherically bent Si or Ge Bragg analyzer with a radius of 1 m or 2 m and a cylindrically bent Laue analyzer with a radius of 1.4 m. Note that the distance between the sample and the analyzer is close to the radius for the Bragg analyzer but it is significantly shorter for the Laue analyzer, 1.2 m.^1^ The 〈111〉 orientation was utilized for the Bragg analyzers. Once the Bragg angle was fixed at 89.5°, the 555, 777, 888, 999 and 11 11 11 reflections were successively observed when *E* was scanned from 9.9–21.8 keV. For the Laue analyzer, the 〈110〉 orientation was utilized. When the Bragg angle was fixed at 30°, the 440, 660 and 880 reflections were observed at *E* = 12.8, 19.5 and 25.8 keV. Owing to practical limitations, to test the Laue analyzer the undulator gap was set to generate the first harmonics at *E* = 6.53 keV. The strong beam was available at *E* = 19.6 keV whereas there were only the weak beams at *E* = 12.8 keV and 25.8 keV. Nonetheless, this is not an issue for the test as long as the photon flux is correctly monitored.

Table 1 ▸ indicates the parameters for the analyzers used in the test. The Si or Ge Bragg analyzers are glued onto the quartz glass substrates with a 1 m- or 2 m-radius curved surface, while the Laue analyzer has no substrate. The base of the triangular wafer was clamped by aluminium blocks while the apex was displaced by the remote actuator, facilitating a continuous change of the radius. The detector for the Bragg analyzers was a photon-counting, Si diode point detector (5 mm diameter and 0.5 mm thickness) while the detector for the Laue analyzer was an NaI scintillation counter (50 mm × 50 mm surface and 3 mm thickness). The scattering angle was adjusted so that the same momentum *Q* was probed while scanning *E* from 9.9 keV to 25.8 keV. The solid angle for photon acceptance for the Bragg analyzers was determined by an aperture of the mask in front of the analyzers. Circular masks of 75 mm- and 85 mm-diameter openings was used for 1 m- and 2 m-radius analyzers, respectively, corresponding to acceptance solid angles of 4.4 × 10^−4^ sterad and 1.4 × 10^−4^ sterad. On the other hand, the acceptance solid angle for the Laue analyzers was defined by a rectangular mask between the sample and the analyzer. The mask was 0.75 m from the sample and the opening was 10 mm × 20 mm (V × H), giving 2.8 × 10^−5^ sterad. The aperture could be opened, but the energy resolution would substantially deteriorate. Therefore, we kept it at 10 mm × 20 mm throughout the test. It remains an issue to be addressed in future of how to make the aperture larger while maintaining the energy resolution at 1–2 eV.

**Figure 1:**
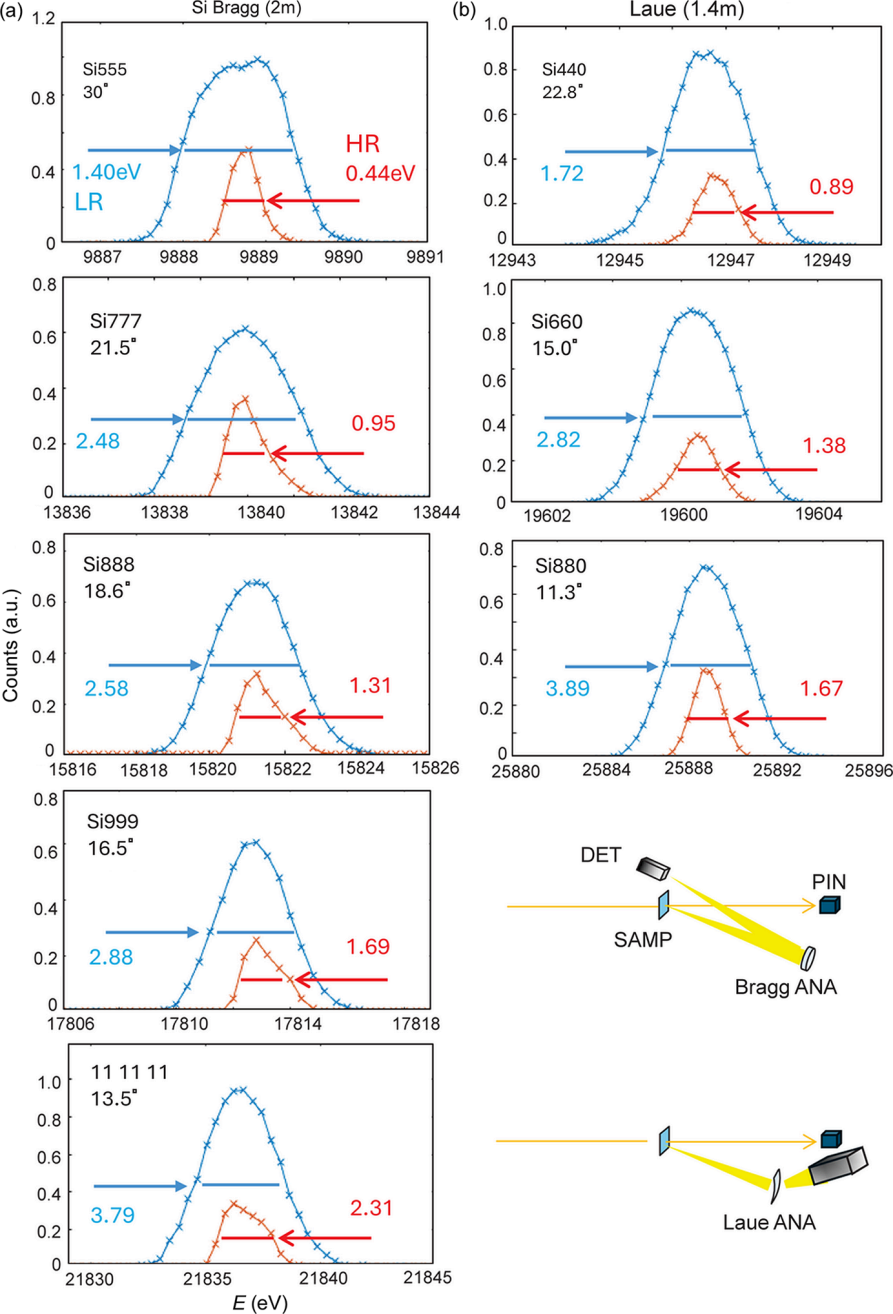
Energy profiles of the elastic scattering on SiO_2_ glass measured by the (*a*) 2 m Si Bragg analyzer and (*b*) 1.4 m Laue analyzer. The insets indicate the experimental geometries.

**Figure 2:**
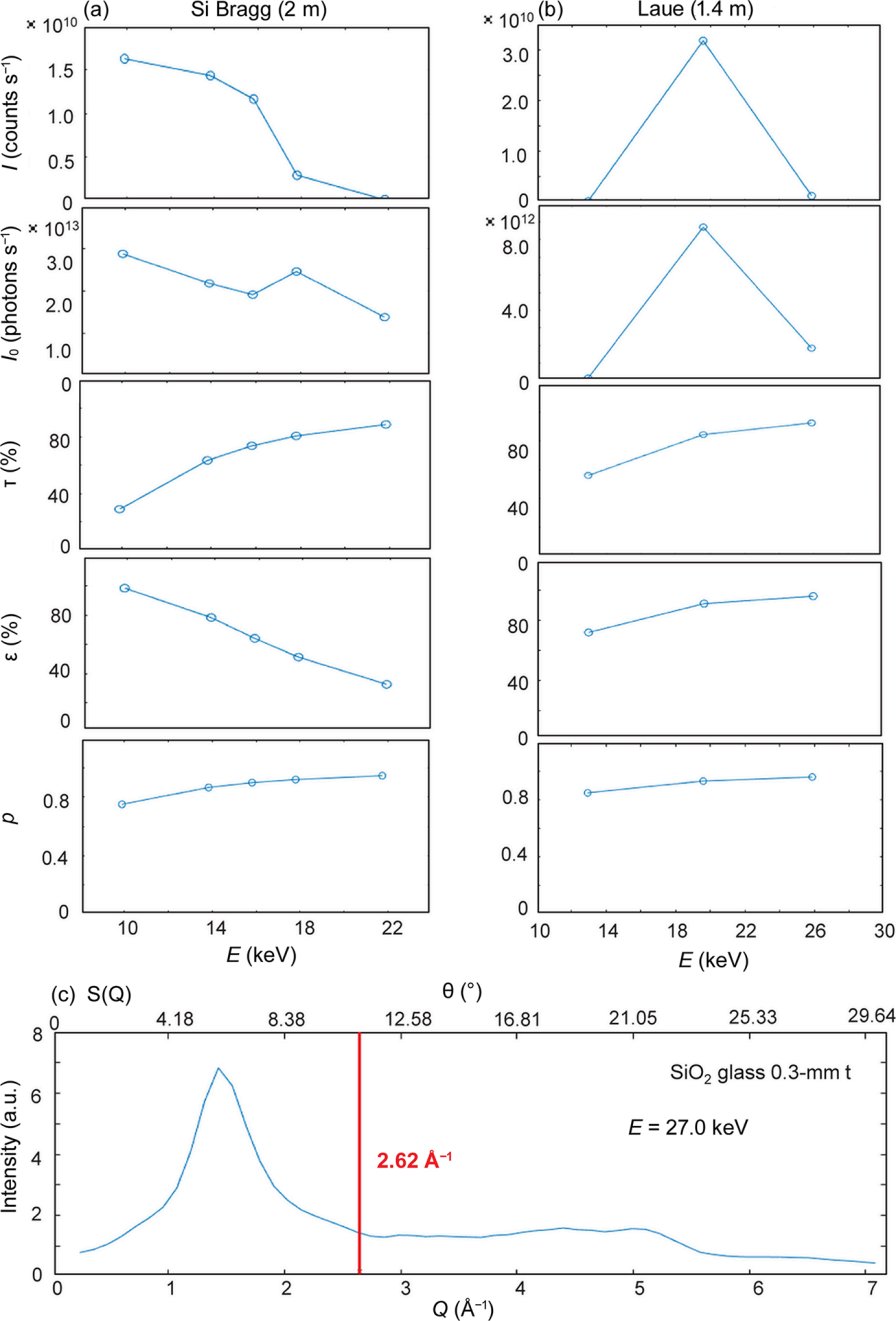
*I* is the integrated intensity of the elastic scattering, *I*_0_ is the incoming photon flux, τ is the transmittance through the sample and the air/helium path, ε is the quantum efficiency of the detector, and *p* is the polarization factor. Transmittance through the Be or Al windows is included in ε. (*a*) Bragg analyzer. (*b*) Laue analyzer. (*c*) *S*(*Q*) of SiO_2_ glass measured by the Laue analyzer with 27 keV photons (0.17 Å^−1^ resolution in *Q*-space). The reflectivity tests were conducted at *Q* = 2.62 Å^−1^.

Fig. 1 ▸(*a*) shows the energy profiles of the elastic scattering measured by the 2 m-radius Si Bragg analyzer and Fig. 1 ▸(*b*) shows those by the 1.4 m Si Laue analyzer. The notation LR stands for measurements taken with a beam from the Si 111 DCM. The Si 111 reflection has a bandpass mostly larger than those of the analyzers. The HR notation is for measurements taken with the Si 220 four-bounce high-resolution monochromator, which generally has a bandpass that is narrower than those of the analyzers. The reflectivity ρ here is defined as (see Appendix *A*[App appa]) 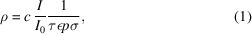
where *I* is the integrated intensity of the elastic scattering, *I*_0_ is the incoming photon flux, τ is the transmittance through the sample and the air/helium path, ε is the quantum efficiency of the detector (taking the transmittance through the Be or Al windows into consideration), and σ is the solid angle for the photon acceptance as already discussed above. *I* and *I*_0_ are observable whereas τ and ε can be calculated using the absorption coefficients. Assuming a perfect in-plane polarization of the incoming beam, the polarization factor *p* is given as (1 + cos2Θ)/2, where Θ is the scattering angle. The energy dependence for each parameter is shown in Fig. 2 ▸. The constant *c* is a normalization factor.

**Figure 3:**
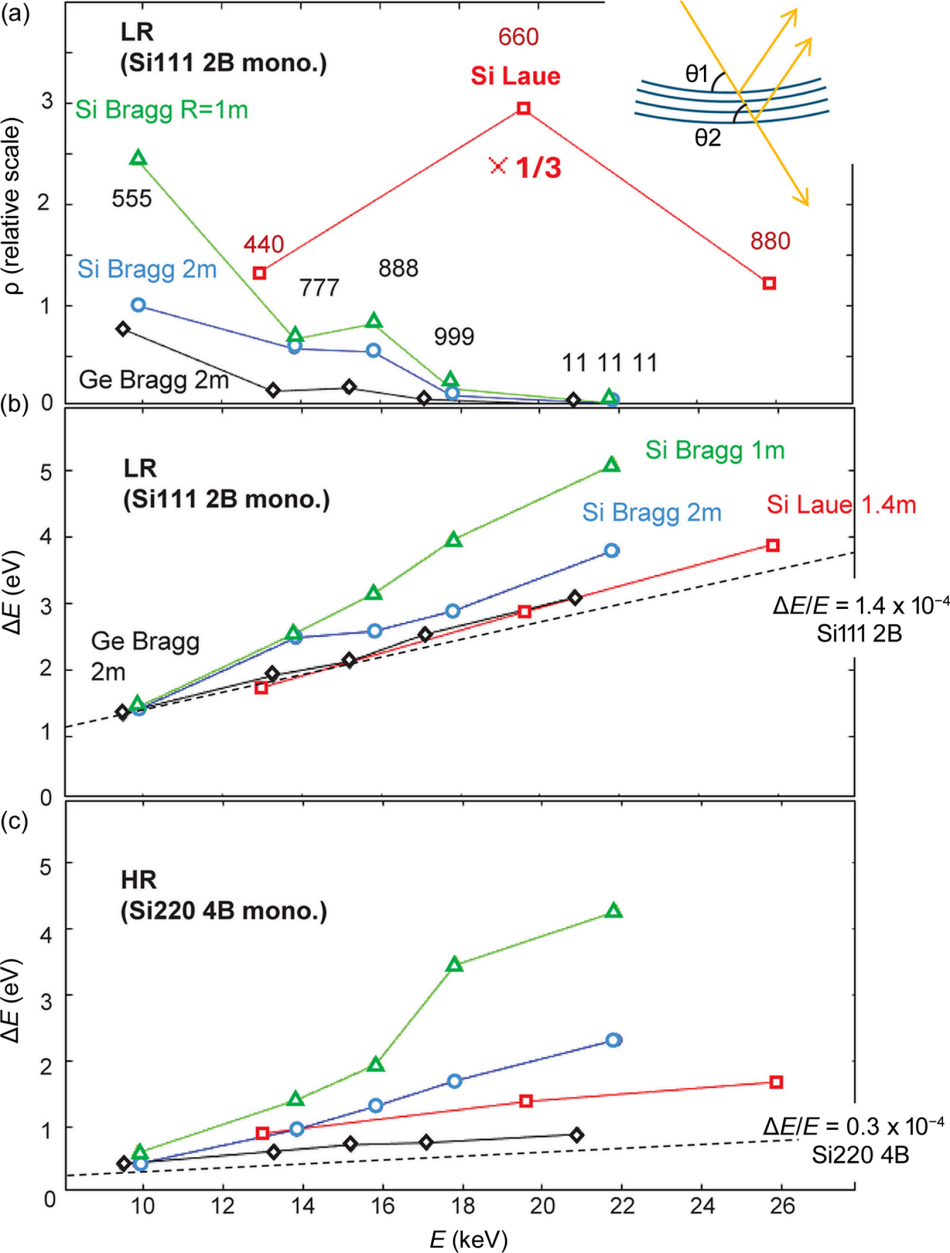
Energy dependence of the (*a*) reflectivity ρ and (*b*)–(*c*) energy resolutions Δ*E*. The circles and triangles represent the 2 m- and 1 m-radius Si Bragg analyzers, respectively, and the diamonds represent the Ge Bragg analyzer. The squares represent the 1.4 m-radius Si Laue analyzer. The scale is normalized such that ρ = 1 for the 2 m Si 555 reflection. LR stands for measurements taken with a beam from the Si 111 DCM. The Si 111 reflection has a bandpass mostly broader than those of the analyzers. HR is for measurements taken with the Si 220 four-bounce high-resolution monochromator, which generally has a bandpass narrower than those of the analyzers. The monochromator contributions Δ*E*_MON_ to the resolution are shown by broken lines in (*b*) and (*c*). Note that Δ*E*_MON_ and the analyzer contribution Δ*E*_ANA_ cannot be linearly summed for most cases: for example, Δ*E*_TOT_^2^ = Δ*E*_MON_^2^ + Δ*E*_ANA_^2^ if they have Gaussian shapes.

Fig. 3 ▸(*a*) displays the reflectivity ρ as a function of photon energy. Here, *c* is determined such that the reflectivity of the 2 m-radius Si 555 Bragg analyzer is 1. Similar experiments were conducted with a 1 m-radius Si〈111〉 Bragg analyzer (triangles in Fig. 3 ▸) and a 2 m-radius Ge〈111〉 Bragg analyzer (diamonds). The reflectivity of the Laue analyzer in *E* = 10–30 keV is excellent. The reflectivity of the Bragg analyzers decrease as *E* increases. The 888 reflections appear somewhat stronger than the others as this is an even-number reflection, which generally has larger scattering structure factors. The reflectivity of the Ge analyzer is lower than that of Si because Ge has a larger absorption coefficient. The Ge *K*-edge is around 11 keV. Therefore, in the energy range above this, the reflectivity is particularly low.

Regarding the energy resolution, the Ge analyzer shows the narrowest width while the Bragg Si shows the widest. The Laue analyzer is between these two. Based on the lamellar model (Erola *et al.*, 1990 ▸), those behaviors can be understood as follows. In a bent crystal, the lattice parameter and the incident angle to the diffraction plane vary as a function of the position from the front surface along the thickness directions (see inset in Fig. 3 ▸) (Suortti *et al.*, 1986*a* ▸). This variation is why bent crystals have a large bandwidth and show a larger (integrated) reflectivity (Suortti *et al.*, 1986*a* ▸,*b* ▸; Pattison *et al.*, 1986 ▸). Nonetheless, Ge has a stronger absorption, so that only diffraction planes near the front surface contribute to the reflections. Therefore, the Ge analyzer shows the narrowest width and the lowest intensity. In a similar way, it can be understood why the 1 m-radius Si Bragg analyzers has a higher (integrated) reflectivity than that of the 2 m-radius ones. This is because of the larger difference between the Bragg angles near the front surface (θ_1_ in Fig. 3 ▸ inset) and those in the interior (θ_2_). Note that the reflectivity in Fig. 3 ▸(*a*) is already corrected for the difference of the solid angles and thus the difference in the reflectivity solely arises from the increase (or decrease) of the bandwidth in the energy. In the Laue analyzer, the effects attributed to the variation in the lattice parameter and the rotation of the diffraction plane can be compensated (Erola *et al.*, 1990 ▸). The degree of compensation can be controlled by the magnitude of the asymmetric cut. This fact was already discussed in a previous report (Hiraoka *et al.*, 2013 ▸). All of the Laue crystals used in this experiment have a 1° asymmetric cut.

**Figure 4:**
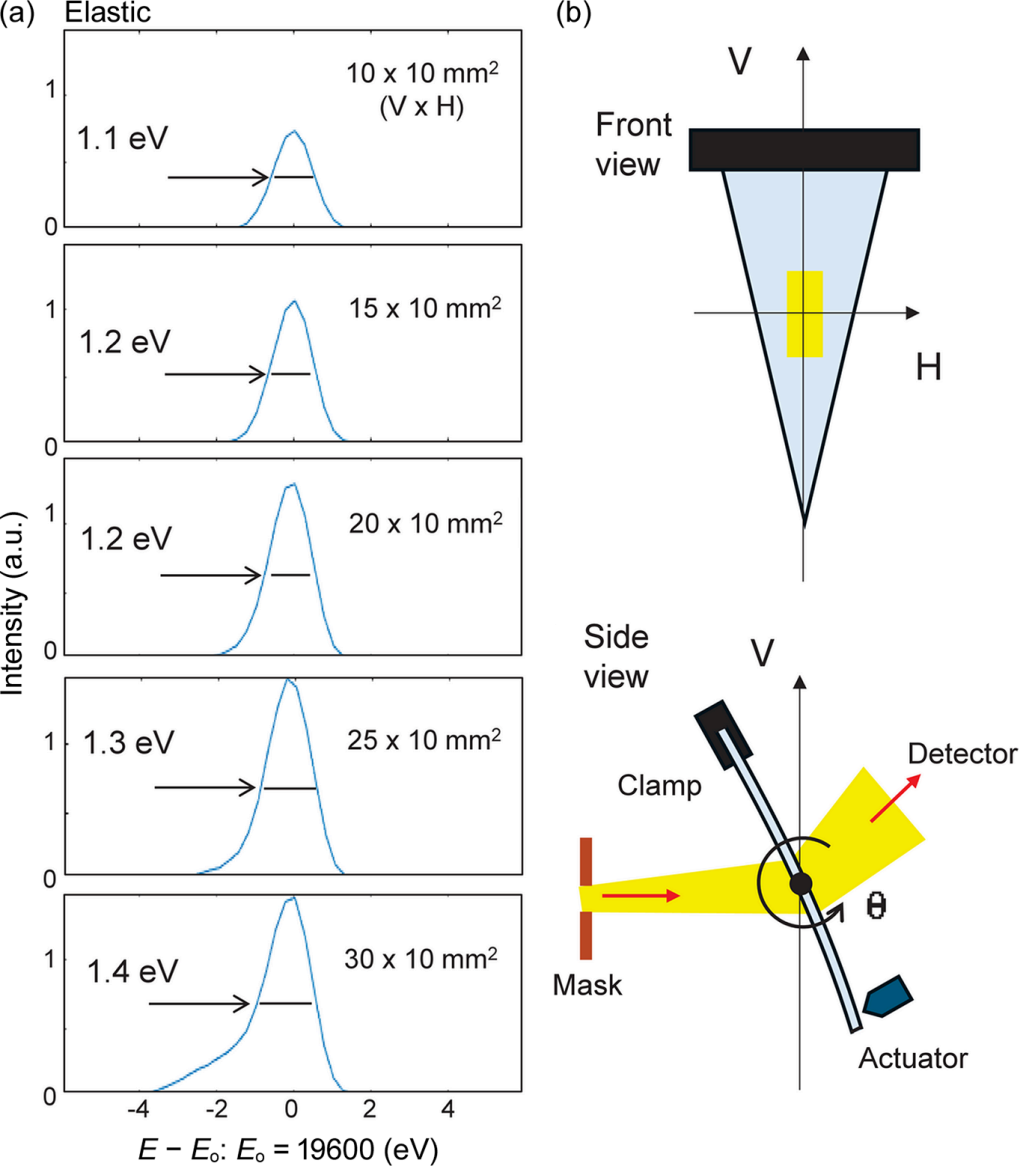
Energy profile of the elastic line measured by the Laue analyzer on SiO_2_ glass as a function of aperture size (*a*), and analyzer layout (*b*).

As mentioned above, Laue analyzers currently perform well when only a limited area is used with a mask. Fig. 4 ▸ shows the elastic line profile as a function of the aperture size. As the aperture size increases, the line profile broadens and a tail becomes more prominent, indicating a deterioration in the resolving power. Currently, a 10–20 mm × 20 mm (V × H) aperture is commonly used. A horizontal opening also deteriorates the resolution, but its effect is less significant compared with the vertical (not shown). Therefore, using a rectangular crystal along with a larger surface detector may increase the active area and enhance the intensity. Nonetheless, in a preliminary test conducted earlier, a resolution better than 2 eV was not achieved with a rectangular crystal. The exact reason for this remains unknown; it could be due to an inappropriate design of the crystal bender or poor quality of the crystal itself. Since then, triangular crystals have been used.

## 3. HERFD-XAS measurements on 4*d* transition metals with Laue analyzers

HERFD-XAS draws much attention from scientists studying catalysts because it can be a powerful tool to find the active site and to clarify the reaction process (Hung *et al.*, 2018 ▸; Guo *et al.*, 2020 ▸; Bai *et al.*, 2021 ▸; Feng *et al.*, 2024 ▸; Matsuyama *et al.*, 2024 ▸). Conventional XAS, whether in transmission or fluorescence mode, suffers from the considerable broadening effects due to the core-hole lifetime, unless the core is shallow (*E* ≤ 1 keV). In HERFD-XAS, one of several fluorescence lines emitted from the sample is focused, and the incident photon energy is scanned across the absorption edge by recording a peak (not integrated) intensity of the fluorescence (Hämäläinen *et al.*, 1991 ▸; Bauer, 2014 ▸). This suppresses the lifetime broadening, allowing fine structures to be easily identified. Currently, HERFD-XAS is often performed on the *K*-edge in 3*d* transition metal elements or *L*-edges in the 5*d* elements because they have emission lines around 10 keV and thus widely used Bragg analyzers work well at this energy. On the other hand, although the 4*d* transition metals include many important elements for catalysis or other energy materials, such as Mo, Rh, Pd and Ag, there are far fewer reports for HERFD-XAS on these elements (Lima *et al.*, 2013 ▸). They have emission lines around 20 keV and it is more difficult to achieve a sufficient intensity and resolution at this energy by Bragg analyzers. The Laue analyzer has a great advantage for HERFD-XAS studies on 4*d* transition metals.

**Figure 5:**
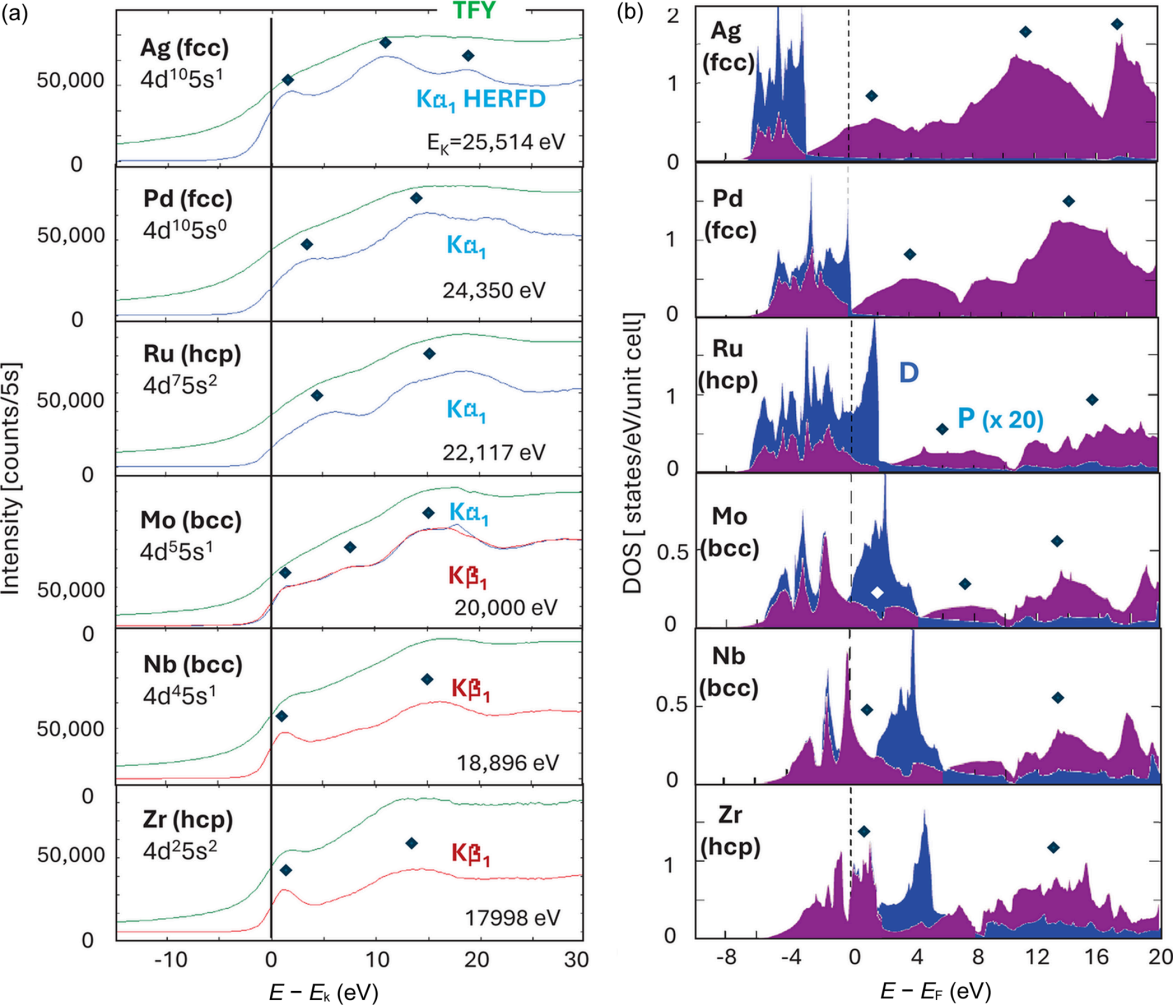
(*a*) HERFD- and TFY-XAS spectra on 4*d* transition metals, and (*b*) partial densities of states calculated by band theory.

Fig. 5 ▸(*a*) shows the *K*-edge HERFD-XAS spectra of Zr, Nb, Mo, Ru, Pd and Ag thin films (∼20 µm) measured by the Si 660 Laue analyzer. They are compared with the total fluorescence yield (TFY) XAS spectra measured by an Si PIN diode simultaneously. The incident photon energy was scanned using the Si 220 four-bounce HRM. During the scans, the peak intensities of the *K*β_1_ lines were monitored for Zr and Nb foils whereas the *K*α_1_ lines were monitored for Ru, Pd and Ag foils. For Mo, both lines were monitored to ensure consistency. The overall energy resolution of the instrumentation was 1–2 eV. The energy resolutions evaluated at each edge are summarized in Table 2 ▸.

The lifetime of the 1*s* core hole in 4*d* transition metals is substantially short, and thus the broadening effect is significant. The effect is larger as the atomic number increases. For Pd and Ag, it can be as large as 10 eV. Because of this, in TFY-XAS it is difficult to find the difference among late 4*d* elements in particular. In contrast, in HERFD-XAS, such a broadening is largely suppressed, and clear differences are readily recognized. The features in HERFD-XAS agree well with the distribution of the partial density of states of *p*-symmetry (*p*-DOS) calculated by band theory.^2^ Generally, 4*d* transition metals have larger spin–orbit couplings than 3*d* transition metals and, therefore, their spectra comprise some *d*-DOS in valence bands. However, it is clear from comparing the HERFD-XAS spectra and the *d*-DOS in Fig. 5 ▸ (note the *p*-DOS is multiplied by a factor of 20) that such a contribution would not be major. This conclusion agrees with the past report (Lima *et al.*, 2013 ▸).

## 4. Comparison of Raman features measured by Bragg and Laue multiple analyzer spectrometers

When performing XRS experiments, an interesting question is whether the Bragg spectrometers operating at *E* ≃ 10 keV or the Laue spectrometer at *E* ≃ 20 keV will produce better quality data in terms of the intensity, energy resolution and other aspects. The answer depends on the experimental conditions, such as the light source, the optics (*e.g.* monochromator and mirrors), the number and size of the analyzers, and the type of detector used. For an example, we compare XRS spectra measured in BL12XU, SPring-8. Here, beams of similar intensities and the same focal sizes are available at*E* = 10 keV and 20 keV (2 × 10^13^ photons s^−1^ versus 1 × 10^13^ photons s^−1^ in a 15 µm × 30 µm focus). Furthermore, a multiple-analyzer spectrometer equipped with 9 or 15 Si Bragg analyzers with a 2 m radius and a spectrometer with 5 Laue analyzers with a 1.4 m radius are available.

The advantages of the Bragg spectrometer are as follows. (1) In principle, the reflections of all analyzers can focus onto a point. Therefore, a high density of photons is available in a small area on the detector. This is advantageous for obtaining a high signal-to-background ratio. (2) The Bragg spectrometer is suitable for high-energy-resolution experiments. The influence of the angular variation Δθ on the energy variation Δ*E* is given by Δ*E*/*E* = Δθ/tanθ_B_. θ_B_ closer to 90° produces a higher resolution. (3) For a similar reason, the spectrum is insensitive to the beam fluctuation. Assuming θ_B_ = 89°, a 1 mm beam shift only leads to an energy shift as small as 0.1 eV or less.

The advantages of the Laue spectrometer are as follows. (1) The degree of freedom for the sample environment is high because the detector is very far from the sample, providing a large available space. In addition, sufficient intensity can be obtained even with a small aperture for the scattered photons owing to the high reflectivity per unit solid angle. Also, high *Q* is achievable using high-energy photons even within a limited 2θ range. (2) The transmission geometry is straightforward since high-energy photons have high penetration power. Critical alignment of the sample is not required unlike in the reflection geometry, and a poor surface does not reduce the scattering intensity. (3) The scan range for the scattered photons is wide as a small motion of θ_B_ corresponds to a large Δ*E*. Even a scan over several kiloelectronvolts is possible (Hiraoka *et al.*, 2020 ▸, 2023 ▸; Yang *et al.*, 2020 ▸; Hagiya *et al.*, 2020 ▸). The Bragg analyzers generally operating at very large θ_B_ provide a scan range of only several electronvolts (when θ_B_ ≃ 85°) or several tens of electronvolts (θ_B_ ≃ 80°), and thus the incident photon energy is usually scanned instead of the scattered photon energy in XRS. In addition, access to a larger *Q* range may be advantageous for studies of non-dipolar transitions on the excitation from deep core levels (Yavaş *et al.*, 2019 ▸; Leedahl *et al.*, 2019 ▸).

**Figure 6:**
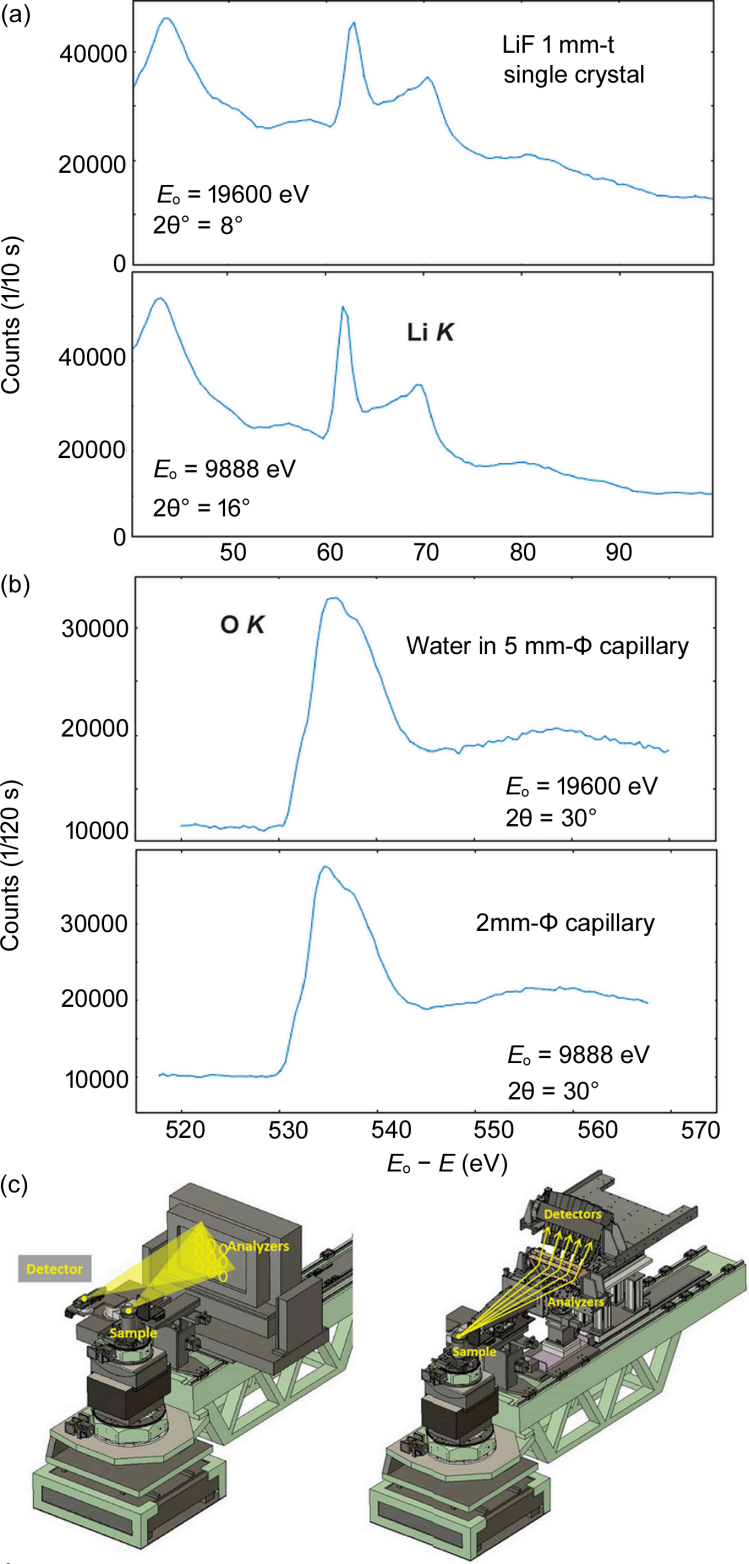
Lithium *K*-edge in LiF (*a*) and oxygen *K*-edge in H_2_O (*b*) Raman features measured by the nine Bragg analyzers (*c*, left) and the five Laue analyzers (*c*, right).

Fig. 6 ▸(*a*) shows the Li *K*-edge feature in LiF measured by the Bragg spectrometer with nine analyzers and by the Laue spectrometers with five analyzers. The scattering angles 2θ = 16° and 8° correspond to the same momentum *Q* = 1.41 Å^−1^ at *E* = 10 keV and 20 keV, respectively. Note that *Q* is the same for the central analyzer but differs slightly for the others. The energy resolutions determined with the elastic scattering are 1.4 eV for both.

In the experiment at 10 keV the beam from the Si 111 DCM was used directly without the HRM, while in the experiment at 20 keV the Si 220 HRM was used after the DCM. The beam intensity in the latter experiment was weaker by a factor of 10 (2 from the light source property and 5 from the HRM). Furthermore, the number of the analyzers (×5/9) and the solid angles for each (×1/4) were smaller in the latter experiment. Nonetheless, the count rates in two experiments are similar. One reason is the larger transmission through the sample of 20 keV photons (×3–4) but the major reason is the high reflectivity of the Laue analyzers.

The Laue spectrometer may be advantageous for studies on energy materials such as batteries and catalysts as these samples are often subject to radiation effects. It would be significant if one could obtain spectra of similar quality with a small number of photons. Furthermore, an absorption coefficient decreases as the photon energy increases. At 10 keV and 20 keV, it differs by a factor of 6–8. Assuming that the dose is proportional to the absorption coefficient × number of photons × photon energy, the dose at 20 keV amounts to only several percent of that at 10 keV.

Fig. 6 ▸(*b*) shows the oxygen *K*-edge features in H_2_O in a capillary with a 2 mm or 5 mm diameter. For both experiments, the scattering angle was 30°, which corresponds to 2.62 Å^−1^ (1.39 a.u.) for *E* = 10 keV and 5.24 Å^−1^ (2.78 a.u.) for 20 keV. If the radius 〈*r*〉 of the oxygen 1*s* orbital is assumed to be *a*_B_/*Z* = 0.125 a.u., where *a*_B_ is the Bohr radius (1 a.u. = 0.529 Å^−1^) and *Z* (= 8) is the nuclear charge, one obtains *Q*〈*r*〉 = 0.174 and 0.348, indicating the dipole transition dominates the XRS spectra for both. The count rate at the maximum near *E* − *E*_0_ = 535 eV was 300 counts s^−1^ at *E* = 10 keV and 280 counts s^−1^ at 20 keV.^3^ Although it is difficult to say which spectrometer is superior because the optimum conditions are different, it is likely that similar-quality spectra can be obtained for both once the experimental conditions are optimized.

## 5. Conclusions

A performance test of spherically bent Bragg analyzers and cylindrically bent Laue analyzers has been conducted. The Bragg analyzer displays an excellent performance at *E* ≤ 10 keV, though the performance gradually deteriorates at higher energies. The Laue analyzer exhibits a superior performance at a high-energy region, indicating that the two types of spectrometers operate in a complementary way. Regarding XES, the Bragg analyzer is generally a better option for measuring the *K* emission lines from the 3*d* transition metals or the *L* emission lines from the 5*d* metals as they are in the *E* ≤ 10 keV range. The Laue analyzers have an advantage in the measurement of *K* emission lines from 4*d* metals, which exist around 20 keV. Concerning the 4*f* rare-earth elements, the *L* emission lines are often measured, but the *K* emission lines have been scarcely studied so far. This vast field remains largely unexplored. XES studies of *K* lines from early rare-earth metals such as La and Ce are among the future targets, which require a spectrometer operating at 30–40 keV. High transmission power of high-energy photons provides us with significant flexibility on the sample environment, such as high temperature, high pressure and *in situ*/*operando* cells.

As for XRS, the spectrometer equipped with nine 2 m-radius Bragg analyzers (operating at *E* ≃ 10 keV) and the spectrometer with five 1.4 m-radius Laue analyzers (at ∼20 keV) show similar performances. They can be switched depending on the purpose. For example, the Bragg analyzers are advantageous if an energy resolution of Δ*E* < 1 eV is required. They are also advantageous for very low count rate (several counts s^−1^) experiments. On the other hand, the Laue analyzers are advantageous for the experiments requiring a high-penetration power and/or a large space around the sample. The current challenge for this analyzer is that only a small portion of the surface can be used to maintain the 1–2 eV resolution. Developing higher-precision benders and using rectangular crystals instead of triangular ones are required to overcome this issue. XRS studies on light-element ingredients in metals, such as carbon in steel, will be an interesting challenge. This would also require high-energy photons.

## Figures and Tables

**Table 1:** Analyzer parameters

	Bragg	Laue
Material	Si/Ge	Si
Surface shape	Circular	Triangular
Dimensions	Ø100 mm	80 × 170 mm†
Thickness	0.5 mm	0.5 mm
Bending	Spherically bent	Cylindrically bent
Radius	1 m/2 m (fixed)	1.4 m (adjustable)
Asymmetric cut	No, symmetric	Yes, 1°
Substrate	SiO_2_ glass, glued	No substrate
Flight path	1.8 m/3.8 m He	1.5 m Air
Aparture of mask	Ø75 mm/85 mm	10 mm × 20 mm‡

† Base × height.

‡ Vertical × horizontal.

**Table 2:** Instrumental energy resolutions in HERFD-XAS measurements on 4*d* absorption edges *E*_*K*_ represents the absorption edge energy and *E*_*K*α/β_ represents the emission energy of *K*α or *K*β lines. The energy resolution is estimated based on the width of the energy profile of the elastic scattering on SiO_2_ glass measured at *E* = 19.6 keV. The energy width of the incident photons Δ*E*_MON_ is evaluated to be 0.59 eV at 19.6 keV by the DuMond diagram analysis. The overall resolution Δ*E*_TOT_ is determined to be 1.38 eV by the measurement. Assuming that Δ*E*_TOT_^2^ = Δ*E*_MON_^2^ + Δ*E*_ANA_^2^, one obtains the resolution of the analyzer crystal Δ*E*_ANA_ = 1.28 eV. In order to estimate the resolution at other energies, one can use the equation Δ*E*_MON_/*E* = ω_D_ tan^−1^θ_B_ constant. Here, ω_D_ is the Darwin width for each reflection and generally ω_D_ ∝ tanθ_B_. For an estimation of Δ*E*_ANA_, one may use the equation (Δ*E*_ANA_/*E*)tanθ_B_ = ω_B_ constant. Here, ω_B_ is the width of the reflectivity curve of a bent crystal and ω_B_ ∝ *R*^−1^. *R* is the bending radius. Generally, ω_B_ ≫ ω_D_ for *R* = 1–2 m crystals.

	*E*_*K*_ (eV)	Δ*E*_MON_ (eV)	*E*_*K*α/β_ (eV)	Δ*E*_ANA_ (eV)	Δ*E*_TOT_ (eV)
Elastic	19600	0.59	19600 (elastic)	1.24	1.37
Zr	17998	0.54	17668 (β_1_)	0.99	1.13
Nb	18986	0.57	18625 (β_1_)	1.11	1.25
Mo	20000	0.60	19588 (β_1_)	1.23	1.37
	20000	0.60	17480 (α_1_)	0.97	1.14
Ru	22117	0.66	19279 (α_1_)	1.19	1.37
Pd	24350	0.73	21177 (α_1_)	1.45	1.63
Ag	25514	0.76	22163 (α_1_)	1.60	1.77

## Footnotes

1 The bending radius is adjustable. This distance of 1.2 m was chosen for geometrical reasons in the experiment.

2 The calculation was performed with a full-potential linearized augmented-plane-wave method based on the local density approximation. *BANDS* code, developed at BL08W, SPring-8, was used.

3 Although the total counts are similar, the spectra measured by the Laue spectrometer appear somewhat noisier. The reason remains unknown. This is perhaps due to cosmic rays or electronic dark noise, as larger area detectors are more prone to detecting them.

## Appendix A The formula for the analyzer reflectivity

Details of the formula for the analyzer reflectivity, given as equation (1), are discussed below. The reflectivity ρ is defined as

Here, *f*_0_ and *f* are the photon flux before and after the analyzer. Using the photon flux in the incoming beam *I*_0_ and the scattering structure factor containing all electron contributions in the scatterer *S*(*Q*)^All^, *f*_0_ can be represented as

where *r*_e_ is the electron classical radius, and τ_0_ is the transmittance through the scatterer (or glass sample) and the helium/air path between the scatterer and the analyzer. The other notations refer to those in equation (1). With the photon flux counted by the detector *I*, *f* is represented as

Here, τ′ is the transmittance through the air/helium path between the analyzer and the detector. Therefore, one finds

where τ = τ_0_τ′. Since the same *Q* (= 2.62 Å^−1^) was probed during the reflectivity measurement, *S*(*Q*)^All^ may be approximated as a constant. Replacing with a scaling factor *c*, one obtains equation (1).

It is debatable whether *S*(*Q*)^All^ can be approximated as a single common parameter because it has an apparent *Q* dependence, while the analyzer has a large *Q* window that varies as a function of the energy and the size of the aperture/mask in front of the analyzer. Around *Q* = 2.62 Å^−1^, where the reflectivity measurements were performed, *S*(*Q*)^All^ exhibits a mild non-linear slope, as seen in Fig. 2 ▸(*c*). By numerical estimation, the errors caused by this effect are estimated to be 6–7% at most when using a 1 m Si Bragg analyzer at 21.8 keV, and 2–3% for most other conditions, where a 2 m Si/Ge Bragg analyzer or Laue analyzer was used. Such errors are relatively small compared with other sources of error, such as the gas concentration in the He path or the individual differences in the analyzer performance.
